# Differences in Chemical Profiles, Phenolic Content, and Antioxidant Activity of *Prunella vulgaris* L. at Different Ripeness Stages

**DOI:** 10.3390/antiox14111270

**Published:** 2025-10-22

**Authors:** Fengqi Liu, Yue Ma, Yufei Liu, Tianze Xie, Liangmian Chen, Wenjiao Lou, Zhimin Wang, Huimin Gao

**Affiliations:** 1 State Key Laboratory for Quality Ensurance and Sustainable Use of Dao-di Herbs, Institute of Chinese Materia Medica, China Academy of Chinese Medical Sciences, Beijing 100700, China; 2 National Engineering Laboratory for Quality Control Technology of Chinese Materia Medica, Institute of Chinese Materia Medica, China Academy of Chinese Medical Sciences, Beijing 100700, China

**Keywords:** *Prunella vulgaris* L., ripeness, fruit-spikes, chemical compounds, phenolic acid, antioxidant activity

## Abstract

*Prunella vulgaris* L. (PV) is a versatile plant with medicinal and culinary applications globally. Its mature fruit-spikes (red-brown) are the primary source of traditional medicine and herbal tea. However, large-scale cultivation and harvesting inevitably leads to the unintended inclusion of immature green fruit-spikes, which are considered substandard medicinal parts. To explore the medicinal and nutraceutical potential of green fruit-spikes, our study systematically compared green and red-brown samples. The distinctions between the two fruit-spikes were characterized by determination of total water-soluble extract content, comprehensive chemical difference analysis, and quantitation of six phenolic acids, including Danshensu, caffeic acid, protocatechuic acid, protocatechualdehyde, salviaflaside, and rosmarinic acid. Additionally, variations in antioxidant activity were evaluated by DPPH and ABTS assays. As a result, 106 compounds were identified from the PV samples. Green spikes exhibited higher total water extract yield and contents of the six phenolic acids than the red-brown ones. Moreover, green samples showed greater accumulations of phenolic acids, flavonoids, and triterpenes. Concomitantly, stronger antioxidant activity was displayed in green spikes in both assay models. Caffeic acid, danshensu, rosmarinic acid, and protocatechualdehyde were identified as major contributors by Pearson correlation analysis. Our findings reveal that green fruit-spikes possess advantages in accumulating specific chemical profiles and exhibiting antioxidant activity, highlighting their untapped pharmaceutical and nutraceutical potential.

## 1. Introduction

*Prunella vulgaris* L. (PV), commonly known as self-heal and Xia-Ku-Cao, is a herbaceous plant with significant nutritional and medical value [1]. PV is widely utilized in Europe, North America, and Southeast Asia. It is consumed as a vegetable, an ingredient in herbal teas and pastries, and in traditional medicine and health supplements [2]. Its medicinal use dates back over 2000 years, with the earliest record found in Shennongbencaojing [3]. The dried fruit-spikes of PV (*Prunella Spica*) are listed in the pharmacopeias of multiple countries, the Chinese medicinal and food homology inventory, and European nutritional supplement registries [4]. PV exhibits antioxidant, anti-hyperlipidemic, anti-microbial, anti-inflammatory, anti-cancer, anti-fibrosis, and anti-osteoarthritic activities [5,6]. It is clinically used to clear liver fire, dissipate nodules, reduce swelling, and improve eyesight [7].

China is the dominant producer of PV, with an output surpassing 20,000 tons in 2025 [8]. Increasing recognition of its pharmacological and nutritional value has driven growing global demand for herbal teas and a steady rise in annual PV consumption. Conventional applications primarily use mature red-brown spikes, while immature green spikes are typically discarded as waste after manual sorting. Nevertheless, large-scale mechanized harvesting inevitably collects substantial quantities of green fruit-spikes. It is therefore essential to explore the medicinal and nutritional potential of PV and promote the comprehensive utilization of PV resources.

PV is rich in phenolic acids, triterpenes and their saponins, flavonoids and their glycosides, phenylpropanoids, and polysaccharides [5,9]. The accumulation of these components exhibits stage-dependent variations, showing dynamic enrichment patterns during fruit-spike maturation [10,11,12]. Notably, the levels of rosmarinic acid, ursolic acid, and oleanolic acid decrease as spikes mature, accompanied by a reduction in antioxidant activity [13,14]. These observations suggest that green spikes may accumulate a broader range of constituents and enhanced antioxidant activity compared to red-brown spikes, and they may hold untapped potential and value for development.

Our team has long been dedicated to the comprehensive development and utilization of PV. Previous works have demonstrated the medicinal and functional food potential of its stems, leaves, and seeds [15,16]. In this study, to investigate the value of green fruit-spikes, a comparative analysis was conducted on simultaneously harvested green and red-brown PV fruit-spikes. Our study evaluated their chemical profiles, total extract yield, content of six key phenolic acids (danshensu, protocatechuic acid, protocatechualdehyde, caffeic acid, salviaflaside, and rosmarinic acid), and antioxidant capacities. This study highlights the advantages of chemical accumulation and antioxidant activity in green PV fruit-spikes and explores their potential for medicinal use or functional food applications. The findings contribute to the reuse of green PV fruit-spikes, reducing waste generated from industrial processing.

## 2. Materials and Methods

### 2.1. Chemicals and Reagents

Acetonitrile and formic acid (HPLC-grade) were all purchased from ThermoFisher Scientific (Waltham, MA, USA); deionized water was obtained by a Milli-Q water purification system (Millipore, Billerica, MA, USA). Other chemicals and reagents were of analytical grade. Chemical standards for qualitative and quantitative analysis, including danshensu and caffeic acid, were purchased from Desite Biological Technology Co., Ltd. (Chengdu, China); protocatechuic acid, protocatechualdehyde, salviaflaside, and rosmarinic acid were purchased from Herbpurify Co., Ltd. (Chengdu, China). Ascorbic acid and 1-diphenyl-2-picrylhydrazine (DPPH) and vitamin C were purchased from Desite Biological Technology Co., Ltd. (Chengdu, China). A T-AOC Kit with the 2,2′-azino-bis (3-ethylbenzothiazoline-6-sulfonic acid) diammonium salt (ABTS) method (S0121) was obtained from Beyotime Biotechnology Co., Ltd. (Shanghai, China).

### 2.2. Plant Material

**Figure 1:**
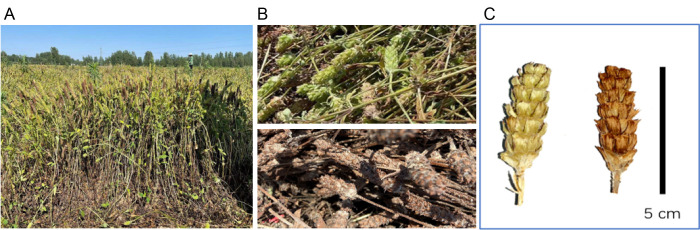
(**A**) The plants of *Prunella vulgaris* L. (PV); (**B**) manually sorted fruit-spikes of PV; (**C**) a close-up photograph of green and red-brown fruit-spikes.

The samples of PV fruit-spikes were collected in mid-May 2023 from Queshan County (32°48′19.2″ N, 114°0′36.8″ E), Zhumadian City, Henan Province. The aerial parts were mechanically harvested and left in the field for sun drying for 2–3 days. Subsequently, the fruit-spikes were separated, manually sorted into green and red-brown ones, and stored in a cool, dry warehouse. Approximately 200 g of random samples was taken for the experimental tests. Botanical authentication was conducted by Prof. Huimin Gao, Institute of Chinese Materia Medica, China Academy of Chinese Medical Sciences, Beijing, China. The PV plant, manually sorted fruit-spikes, and a close-up photograph of green and red-brown fruit-spikes are shown in Figure 1.

### 2.3. Extraction

Since both TCM and herbal teas are conventionally prepared by decoction in water, and 75% ethanol has been shown to yield the highest number of identifiable compounds [17], we prepared both water and 75% ethanol extracts in parallel for chemical analysis.

Dried green and red-brown samples were pulverized and sieved separately. Each powder (1.0 g) was dissolved in 20 mL of solvent and then refluxed for one hour, with solutions cooled down at room temperature, and the weight loss was made up for by solvent. Subsequently, the extracts were centrifuged at 12,000 rpm for ten minutes and filtered through a 0.22 μm filter to obtain the sample solution for phenolic acid detection and antioxidant activity assays. The sample solution was diluted four times for UPLC-MS/MS analysis.

### 2.4. Water-Soluble Extract Determination

The water-soluble extract determination was performed following the guidelines from the *Chinese Pharmacopoeia* (ChP 2020) [18]. The green and the red-brown samples (2.0 g) were extracted with 50 mL water through heating reflux for one hour. After cooling down at room temperature, the solution was weighed again, and the weight loss was made up for with water. Then, via filtering, the filtrate evaporated to dryness and was cooled down to room temperature. The samples for water-soluble extract determination were obtained.

### 2.5. Chemical Analysis by LC-MS/MS

The chemical analysis was performed by an ACQUITY H-class ultra-high-performance liquid chromatography device with a Xevo G2-S QTOF quadrupole high-resolution time-of-flight mass spectrometer (Waters Corporation, Milford, MA, USA). Chromatographic separation was performed on ACQUITY UPLC BEH C_18_ (2.1 × 100 mm, 1.7 µm) (Waters, Milford, MA, USA). The mobile phase was (A) acetonitrile and (B) formic acid/water (1:999, *v*/*v*). The elution gradient program was as follows: 0–6 min, 14–20% A; 6–9 min, 20–34% A; 9–13 min, 34–37% A; 13–13.1 min, 37–60% A; 13.1–20 min, 60–90% A; 20–20.1 min, 90–14% A; 20.1~23 min, 14–14% A. The column temperature was 30 °C. The solvent flow rate was 0.3 mL/min. The sample injection volume was 1 μL.

The analysis was performed by a negative-ion mode using a Q Trap mass with an electrospray ion source (ESI) at a temperature of 120 °C. The capillary voltage was 2.0 kV. The cone voltage was 40 V. The auxiliary spray ionization and desolvation gas was high-purity nitrogen, and the desolvation temperature was 400 °C. The cone gas flow was 50 L/h, and the desolvation gas flow was 600 L/h. The scan range was *m*/*z* 100–1800. The scan time was 0.5 s. The collision voltage was 25–50 eV. And the mass spectrometry data were collected and processed using a MassLynx V4.1 workstation (Waters, Milford, MA, USA).

### 2.6. Detection of Six Phenolic Acids

The quantitative analysis of six phenolic acids was performed by the method established by our team previously [15]. Detection was carried out using an ACQUITY H-class ultra-high-performance liquid chromatograph equipped with ACQUITY UPLC HSS T3 (2.1 × 100 mm, 1.7 µm) (Waters, Milford, MA, USA). The mobile phase was (A) acetonitrile and (B) formic acid/water (1:999, *v*/*v*). The methanol solution of the reference standards included danshensu (0.102 mg/mL), protocatechuic acid (0.00555 mg/mL), protocatechualdehyde (0.00576 mg/mL), caffeic acid (0.0400 mg/mL), salviaflaside (0.0150 mg/mL), and rosmarinic acid (0.207 mg/mL). Gradient elution was performed with 4–8% A in 0–7 min, 8% A in 7–13 min, 8–17% A in 13–14 min, and 17% A in 14–30 min. The column temperature was 35 °C. The detection wavelength was 280 nm. The flow rate was 0.3 mL/min, and the injection volume was 1.0 µL.

### 2.7. Antioxidant Assay

The stock solution of PV fruit-spike extracts (equivalent to 50 mg raw material per mL) was stepwise diluted with water or 75% ethanol to generate medicinal materials with mass concentrations of 10.0, 5.0, 2.50, 1.25, 0.625, 0.313, 0.156, 0.0781 mg/mL. Antioxidant activities were evaluated using two assay methods, DPPH and ABTS. Aliquots of different concentrations of the sample solution (0.5 mL) were added into 3 mL of DPPH–ethanol solution (95%, *w*/*v*, 50 μg/mL). Additionally, 0.5 mL of water or 75% ethanol was added into 3.0 mL of 95% ethanol solution as the blank solution. And 0.5 mL of water or 75% ethanol was added into 3.0 mL of DPPH–ethanol solution as the control solution [19]. After one hour, the absorbance was measured at 517 nm using a UV759CRT UV–visible spectrophotometer (Yoke Instrument, Shanghai, China) [20].

The ABTS assay determined the capacity for radical quenching through electron transfer. The ABTS assay was measured according to the instruction method of the T-AOC Kit with the ABTS method (S0121, Beyotime Biotechnology, Shanghai, China). After mixing with the ABTS working solution, the absorbance was recorded at 414 nm using a microplate reader (BMG LABTECH, Offenburg, Germany) [21].

All measurements were performed in triplicate. The DPPH or ABTS quenching effect was calculated as follows:

DPPH or ABTS scavenging effect (%) = [1 − (A sample − A blank)/A control] × 100%

### 2.8. Statistical Analysis

Results are expressed as mean ± standard deviation (SD). Normality and homoscedasticity tests were performed. Then, variance analyses were performed using a one-way ANOVA followed by Tukey’s HSD post hoc test or Dunnett’s T3 multiple-comparison test for pairwise comparisons on GraphPad Prism 9 (GraphPad Software, San Diego, CA, USA). The sample size for the biological replicates was three (*n* = 3). A nonlinear fit was conducted on the DPPH and ABTS scavenging effect using GraphPad Prism 9 to obtain the half-maximal effective concentration (EC_50_).

Pearson correlation analysis was performed to investigate the correlation between the content of six phenolic acids and the antioxidant activity of four types of samples, including green and red-brown fruit-spikes extracted using both water and 75% ethanol.

## 3. Results and Discussion

### 3.1. Content of Total Water-Soluble Extracts

To assess the overall chemical distinctions between green and red-brown PV fruit-spikes, both sample types were extracted with water following the protocols outlined in the ChP 2020. A significant difference (*p* < 0.001) was observed in the total content of water-soluble extracts: green fruit-spikes yielded 25.6 ± 0.557%, whereas red-brown fruit-spikes contained 16.3 ± 0.213% (mean ± SD, *n* = 3). These results indicate that the water-soluble extract content of green samples is over 1.5 times higher than that of red-brown ones. This observation aligns with previous reports documenting a continuous decrease in numerous components during maturation [10,11,12] and suggests a correlation between visual color characteristics and water-soluble extract levels: a higher level of total component accumulation was found in green fruit-spikes, while a lower level was found in red-brown ones.

### 3.2. LC-MS/MS Analysis

#### 3.2.1. Identification of Components by LC-MS/MS

**Figure 2:**
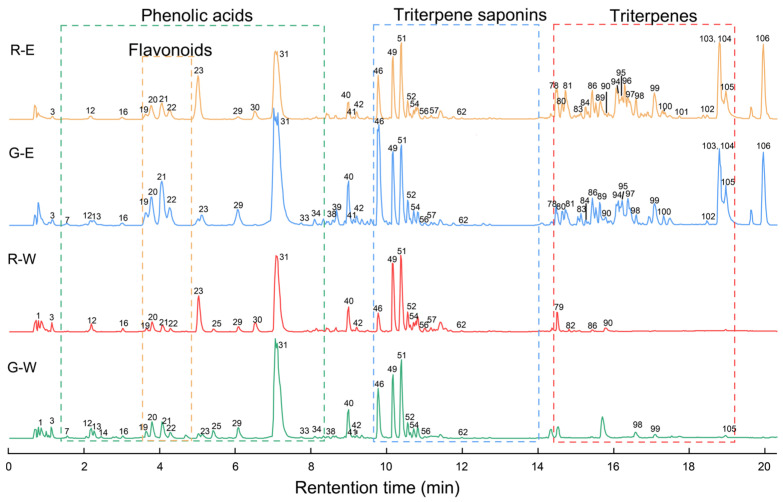
Typical total-ion chromatograms of green and red-brown samples. G, green fruit-spikes; R, red-brown fruit-spikes; W, water extract; E, 75% ethanol extract. The serial numbers presented in the figure align with the serial numbers of the compounds listed in Table 1.

To clarify the components contributing to the significant difference in total extract yields of the two fruit-spikes, we first comprehensively identified all components, followed by differential analysis. A total of 106 components were identified in the water and 75% ethanol extracts of both green and red-brown spikes by UPLC-MS/MS. Compound identification relied on retention times, UV spectra, quasi-molecular ions, and adduct ion peaks from MS and characteristic MS/MS fragment ions. These components were segregated into distinct classes by retention time: phenolic acids (0.5–8.3 min), flavonoids (3.6–5.2 min), triterpene saponins (8.4–14.3 min), and triterpenes (14.5–20.0). Identification was validated against reference data, online databases such as PubChem, ChemSpider, and SciFinder, and our previous report [15]. Typical total-ion chromatograms of green and red-brown fruit-spikes are shown in Figure 2, and the full list of identified components is provided in Table 1.

The PV fruit-spikes exhibited a notable enrichment of phenolic acids, with rosmarinic acid (*m*/*z* 359.0770) being the base peak. Salviaflaside followed (521.1288 Da), differing from rosmarinic acid by an additional glucose molecule (162.0520 Da). Peaks at *m*/*z* 137.0250 and 153.0202 were identified as protocatechualdehyde and protocatechuic acid, respectively, with a mass difference of an oxygen atom (15.9952 Da) between them. Additionally, caffeic acid and dihydrocaffeic acid yielded signals at *m*/*z* 179.0347 and 181.0508, respectively, differing by a H2 unit (mass difference of 2.0161 Da). Danshensu was identified at *m*/*z* 197.0452, exhibiting additional H_2_O compared to caffeic acid.

The compounds salvianolic acid B and isosalvianolic acid B were characterized by their molecular ions at *m*/*z* 717.1435, accompanied by fragment ions at *m*/*z* 519.0933, 339.0566, and 295.0604, corresponding to the sequential loss of danshensu, caffeic acid, and CO_2_ molecules. Based on their polarity order in the literature [22], the peak observed at 9.13 min was identified as salvianolic acid B, while the peak observed at 9.22 min was identified as isosalvianolic acid B. Furthermore, their isomers also generated a molecular ion at *m*/*z* 717.1435 with fragment ions at *m*/*z* 519.0933 and 339.0566. Given the order of the peaks and the presence of these fragment ions, this suggested that peak 26 potentially represents isosalvianolic acid E, while peak 47 may correspond to salvianolic acid E/L [23].

**Figure 3:**
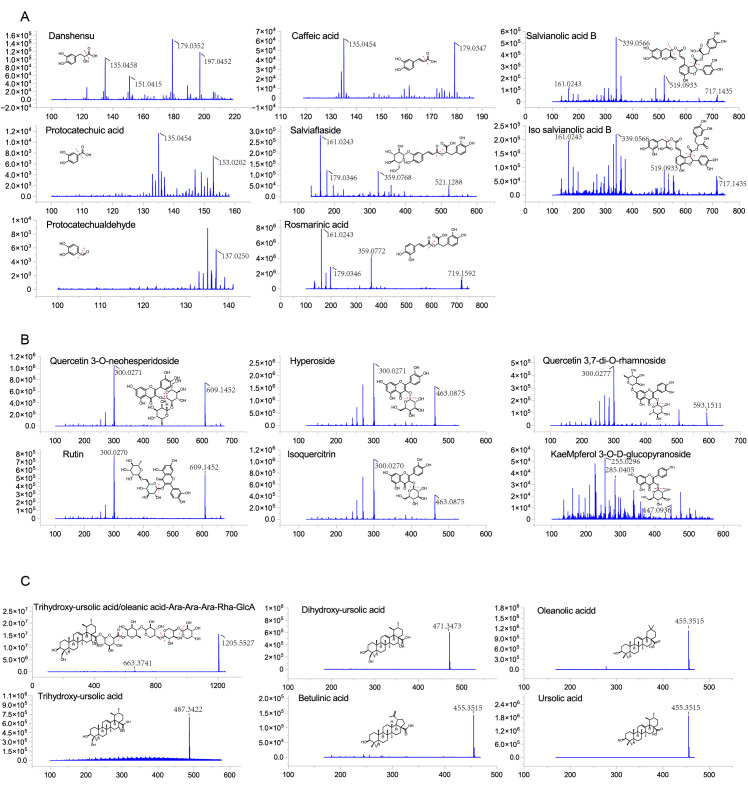
The mass spectra and structures of (**A**) rosmarinic acid, salviaflaside, protocatechuic acid, protocatechualdehyde, caffeic acid, danshensu, salvianolic acid B, and isosalvianolic acid B; (**B**) quercetin 3-O-neohesperido-side, rutin, hyperoside, isoquercitrin, quercetin 3,7-di-O-rhamnoside, and kaempferol 3-O-*D*-glucoside; (**C**) typical triterpenoid saponin, dihydroxy triterpene, trihydroxy triterpene, betulinic acid, oleanolic acid, and ursolic acid.

Salvianolic acid A was identified based on its molecular ion at *m*/*z* 493.1137, along with the loss of a molecule of caffeic acid resulting in a fragment ion at *m*/*z* 313.0717, and the fragment ion at *m*/*z* 295.0609 could be attributed to either the loss of a molecule of danshensu or sequential dehydration following removal of caffeic acid [24]. The identification of chlorogenic acid was based on its molecular ion at *m*/*z* 353.0876, accompanied by fragment ions at masses of 191.0561, 179.0355, 135.0459, and 161.0244 [25]. Characteristic mass spectra and chemical structures of the major phenolic acids are shown in Figure 3A.

Several flavonoids were detected in PV fruit-spikes based on their fragment ions. Peaks eluting at 3.62, 3.81, 4.08, 4.28, and 5.19 min were identified as flavone glycosides, characterized by fragment ions at *m*/*z* 300.0270 and 301.0334. The components eluting at 3.62 and 3.81 min (molecular ion at *m*/*z* 609.1452) were assigned to quercetin 3-O-neohesperidoside and rutin, respectively. Relative to quercetin, these compounds contain an additional glucose and a rhamnose. Peaks observed at 4.08 and 4.28 min, with a molecular ion at *m*/*z* 463.0875, indicated a single additional glucose or galactose. These peaks were identified as hyperoside and isoquercitrin, respectively. Furthermore, the compound at 5.19 min (molecular ion at *m*/*z* 593.1511) was characterized as quercetin 3,7-di-O-rhamnoside, corresponding to quercetin with two rhamnoses. Additionally, a fragment ion at *m*/*z* 447.0936 was identified as kaempferol 3-O-*D*-glucoside or homoorientin due to its mass being 162 Da higher than that of kaempferol (*m*/*z* 285.0405) [26,27]. Representative mass spectra and chemical structures of the major flavonoids are shown in Figure 3B.

Several triterpene saponins were found in the samples, with a notable elution range between 8.4 and 14.3 min. These saponins were classified into oleanane- and ursane-type. Previous studies have reported their association with disaccharides or trisaccharides [28,29]. Notably, our analysis revealed, for the first time, abundant polyglycosylated derivatives containing four to six linked sugars. Among these triterpene saponins, compounds yielding a fragment ion at *m*/*z* 487.3422 were assigned to skeletons derived from trihydroxy-ursolic acid or trihydroxy-oleanolic acid, while those with fragment ions at *m*/*z* 471.3473 were attributed to dihydroxy-ursolic acid or dihydroxy-oleanic acid. Furthermore, the oligosaccharide chain was predominantly composed of glucuronic acid, glucose, arabinose, galactose, and rhamnose. For instance, the peak at 10.39 min was identified as trihydroxy-ursolic acid/oleanic acid-Ara-Ara-Rha-Ara-GlcA, based on its molecular ion at *m*/*z* 1205.5527 and fragment ions at *m*/*z* 1073.5099, 941.4502, 795.4188, 663.3741, and 487.3427. These fragments corresponded to subsequent losses of three arabinoses (132.0428 Da each), one rhamnose (146.0597 Da), and one galacturonic acid (176.0314 Da), with the ion at *m*/*z* 487.3427 confirming trihydroxy ursolic acid or trihydroxy oleanolic acid. Representative mass spectra and chemical structures of typical triterpenoid saponins are shown in Figure 3C.

In total, 12 triterpenes were identified in the fruit-spike samples, including betulinic acid, oleanolic acid, ursolic acid, 5 dihydroxy derivatives, and 4 trihydroxy derivatives. Both oleanane- and ursane-type triterpenes exhibited rare fragment ions in the ESI. According to the elution order and the molecular ion at *m*/*z* 455.3515, the peak at 18.50 min was identified as betulinic acid, the peak at 18.83 min was identified as oleanolic acid, and the peak at 19.00 min was identified as ursolic acid [30]. Additionally, dihydroxy derivatives were identified by the molecule ion at *m*/*z* 471.3473, and trihydroxy derivatives by that at *m*/*z* 487.3422. Representative mass spectra and chemical structures of betulinic acid, oleanolic acid, and ursolic acid are shown in Figure 3C.

Additionally, two organic acids were identified. The peak at 18.80 min (molecule ion at *m*/*z* 277.2166) corresponded to linolenic acid, while the peak at 19.96 min (molecule ion at *m*/*z* 279.2322) was assigned to linoleic acid, differing from linolenic acid by two hydrogen atoms. These two fatty acids were consistent with previous reports identifying them as major components in PV seeds [31].

Although numerous compounds were identified in the two types of spikes, several isomers with unidentified structures are mentioned in our results. Additionally, a notable finding was the observation of triterpenoid saponins with 4–6 sugars, exceeding the previously reported range of 2–3 sugars. However, due to the limitations of mass spectrometry, it is impossible to accurately predict the conformations for certain compounds. In particular, the sugar linkage sequences of the saponins could not be fully elucidated. Therefore, the isolation and structural identification of these components would be our primary objective in future research.

#### 3.2.2. Comparison Compounds Between Green and Red-Brown Fruit-Spikes

**Figure 4:**
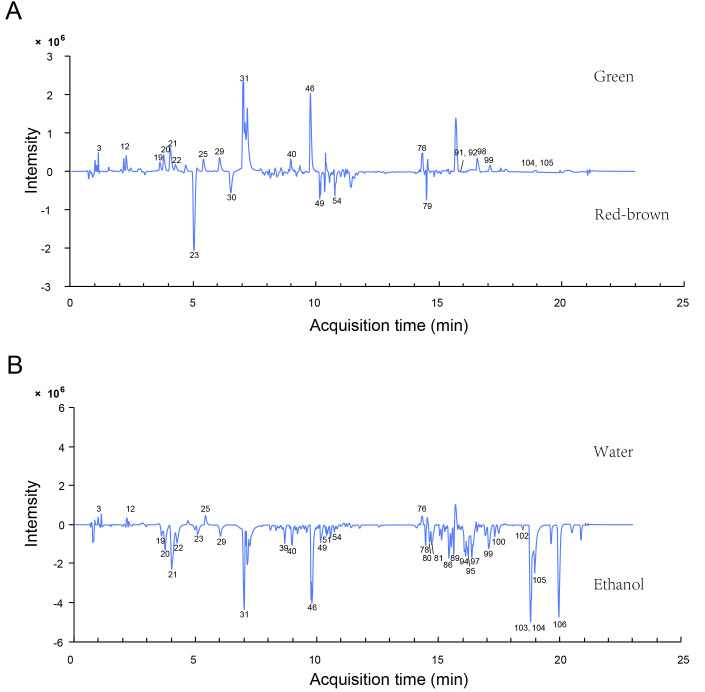
Differences between green and red-brown fruit-spike samples and between extraction solvents. (**A**) Subtraction of the base peak intensities of the water extracts of a green (above) and a red-brown (below) sample; (**B**) subtraction of the base peak intensities of the green sample extracted using water (above) and 75% ethanol (below). The serial numbers presented in the figure align with the serial numbers of the compounds listed in Table 1.

To compare the chemical composition differences between green and red-brown fruit-spikes, the total-ion chromatogram (TIC) signals of water extracts from green and red-brown fruit-spikes were subtracted (Figure 4A). Generally, the upper chromatogram displayed a greater number of peaks than the lower one, demonstrating that most components accumulate at higher levels in green fruit-spikes. This observation aligns with our total extract content measurements, which showed remarkably higher water-soluble extract levels in green samples compared to red-brown ones. Notably, green fruit-spikes were enriched in phenolic acids, triterpenes, and flavonoid glycosides, including rosmarinic acid (31), rutin (20), and hyperoside (21), whereas red-brown samples preferentially accumulated phenolic acid glycosides and triterpenoid saponins, such as salviaflaside (23) and trihydroxy-ursolic acid-Ara-Ara-Ara-Rha-Xyl-GlcA (49).

Our findings reveal a strong association between the fruit-spikes’ developmental stage and chemical accumulation patterns. During this period, phenolic acids and triterpenoids are converted into their corresponding glycosides, while flavonoid glycoside levels decline. This trend is consistent with previous reports showing a peak accumulation of phenolic acids, flavonoid glycosides, and triterpenes at the full-flowering stage, followed by reduced levels during fruit ripening [12,32]. Particularly, key components responsible for PV’s antioxidant and anti-tumor activities, such as rosmarinic acid, ursolic acid, and oleanolic acid, exhibit significant concentration decreases from early to late May during the harvest period [33].

This developmental regulation of metabolite accumulation is linked to the activity of enzymes involved in the biosynthesis of phenolic acids, flavonoids, and triterpenes through the phenylpropanoid pathway [12,13]. Previous studies have shown that enzymes in this pathway reach maximal abundance and activity before fruit-spike emergence, followed by a notable decline as spikes mature into the red-brown stage. Conversely, glycosylation-related enzymes, including ABC transporter subfamily C member 5 and 15, exhibit increased abundance during ripening, driving the conversion of phenolic acids and triterpenes into their glycosylated derivatives [34]. Moreover, UV-B radiation has been reported to enhance glycoside biosynthesis, particularly that of salviaflaside, during the mature-fruiting stage [14]. Our findings demonstrate that green fruit-spikes have advantages in terms of the accumulation of phenolic acids, flavonoid glycosides, and triterpenoids, particularly components with confirmed antioxidant activity such as rosmarinic acid, hyperoside, and oleanolic acid [35,36].

#### 3.2.3. Influence of Extraction Solvents

To evaluate extraction solvents for green spikes, water extracts (used traditionally) and 75% ethanol extracts (previously shown to be optimal [15]) were compared. TIC signal subtraction analysis revealed that 75% ethanol extracts yielded significantly higher levels of most components (Figure 4B). Most flavonoid glycosides, triterpenes and triterpene saponins, and polyunsaturated fatty acids were enriched in 75% ethanol. Key bioactive components such as rosmarinic acid, dedihydro-salvianolic acid B isomer, rutin, hyperoside, oleanolic acid, ursolic acid, linolenic acid, and linoleic acid showed notable better dissolution. Although water extraction is the commonly used method for PV applications, our findings indicate that 75% ethanol offered advantages for dissolving most green fruit-spike components.

### 3.3. Content of Six Phenolic Acids

**Figure 5:**
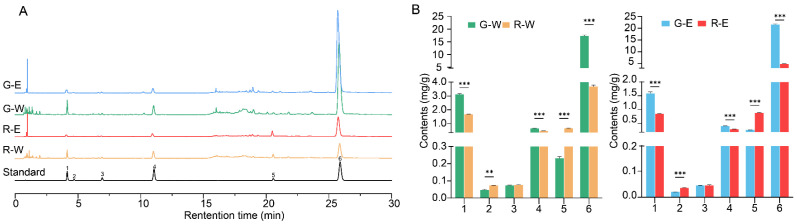
(**A**) Typical UPLC chromatograms of G-E, G-W, R-E, and R-W samples and mixture standards; (**B**) the content of six phenolic acids in samples of G-W, R-W, G-E, and R-E. G, green fruit-spikes; R, red-brown fruit-spikes; W, water extract; E, 75% ethanol extract. 1, danshensu; 2, protocatechuic acid; 3, protocatechualdehyde; 4, caffeic acid; 5, salviaflaside; 6, rosmarinic acid; ** and *** indicate a significant difference according to Dunnett’s T3 multiple-comparison test for protocatechuic acid contents or Tukey’s post hoc test for others, corresponding to *p* ≤ 0.01 and *p* ≤ 0.001.

Phenolic acids, key antioxidants in PV, were quantified to assess the potential medical and nutritional value of green fruit-spikes. Six phenolic acids were selected. Rosmarinic acids and salviaflaside are the primary compounds and quality markers of PV. Caffeic acid, Danshensu, protocatechuic acid, and protocatechualdehyde are recognized as antioxidants in PV [4,34,35]. The contents of the six phenolic acids (mean ± SD, *n* = 3), determined by UPLC with the established method, were compared across sample types and extraction solvents. The green fruit-spikes exhibited a significantly higher total content of six phenolic acids (24.0–21.4%) than the red-brown ones (6.65–6.93%). This result aligns with the higher extract yield and UPLC-MS/MS profiling data, corroborating the chemical advantage of green spikes. Representative chromatograms are shown in Figure 5A, and contents of six phenolic acids are presented in Table 2 and Figure 5B.

Notably, green samples contained significantly higher levels of danshensu (1.59–3.1 mg/g) and rosmarinic acid (17.4–21.6 mg/g) compared to red-brown ones (0.86–1.68 mg/g and 3.68–4.8 mg/g, respectively). A modestly higher level of caffeic acid was also observed in green fruit-spikes (0.42–0.65 mg/g) compared to that in red-brown samples (0.30–0.48 mg/g). These findings indicate that danshensu, rosmarinic acid, and caffeic acid are preferentially accumulated in green spikes, and their contents decrease as the fruit-spikes mature. In contrast, salviaflaside content was significantly higher in red-brown spikes (0.67–0.89 mg/g) than in green ones (0.23–0.28 mg/g). Red-brown samples also showed a slight elevation in protocatechuic acid (0.036–0.073 mg/g vs. 0.02–0.05 mg/g in green spikes), revealing that these two phenolic acids are enriched during maturation. Protocatechualdehyde levels were comparable between the two fruit-spike types, ranging from 0.045 to 0.076 mg/g. These outcomes support the general trend that, as fruit-spikes mature, the content of most phenolic acids declines, while that of their glycosylated derivatives increases.

Additionally, the extraction solvent significantly influenced the concentrations of the six phenolic acids. Both green and red-brown fruit-spikes exhibited the same trend: 75% ethanol extracts yielded a higher total phenolic acid content. Specifically, danshensu, protocatechuic acid, protocatechualdehyde, and caffeic acid were more abundant in water extracts, whereas salviaflaside and rosmarinic acid showed markedly higher levels in 75% ethanol extracts. Notably, the highest content of rosmarinic acid (21.6 ± 0.168 mg/g) was detected in green spikes with 75% ethanol, highlighting their potential as a superior source of this antioxidant [37]. Based on these findings, we intended to evaluate the antioxidant activity of the fruit-spikes.

### 3.4. Antioxidant Activity of Green/Red-Brown Fruit-Spikes

**Figure 6:**
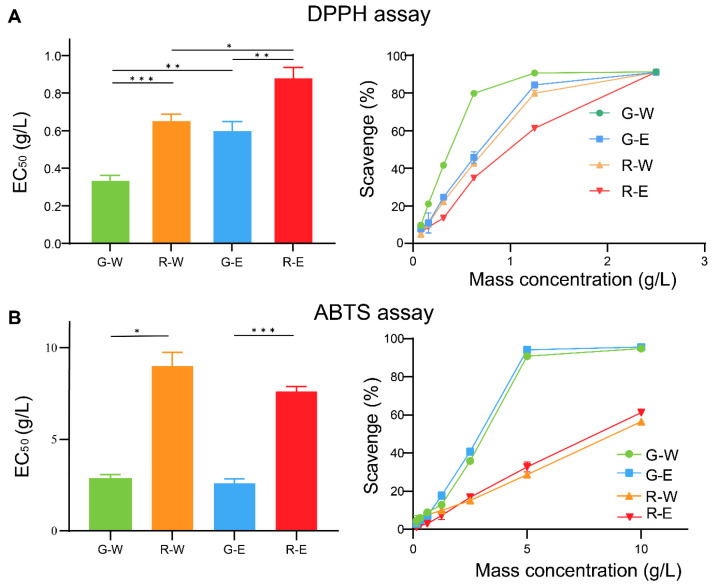
The EC_50_ value and the scavenge of DPPH (**A**) and ABTS (**B**) with G-W, R-W, G-E, and R-E samples. G, green fruit-spikes; R, red-brown fruit-spikes; W, water extract; E, 75% ethanol extract. *, ** and *** indicates a significant difference according to the Dunnett’s T3 multiple comparison test corresponding to *p* ≤ 0.05, *p* ≤ 0.01 and *p* ≤ 0.001, respectively.

The antioxidant activity of the fruit-spike samples was evaluated using DPPH and ABTS assays, which offer complementary mechanisms for a more reliable assessment [38]. The results were assessed by determining the EC_50_ values in Table 3 and Figure 6. The EC_50_ values of the standard controls were 28.59 ± 1.71 μg/mL (Vitamin C, DPPH) and 152.43 ± 13.02 μg/mL (Trolox, ABTS). In both assays, the green fruit-spikes exhibited significantly lower EC_50_ values than the red-brown ones under identical extraction conditions. The results indicate a higher antioxidant activity of green fruit-spikes.

Two antioxidant assays exhibited a consistent trend. Given that the antioxidant activity of phenolic acids primarily involves hydrogen atom transfer [39,40], Pearson correlation analysis was performed between the content of six phenolic acids and the EC_50_ values of DPPH. The results showed a negative correlation for caffeic acid (−0.961), danshensu (−0.977), rosmarinic acid (−0.639), and protocatechualdehyde (−0.596), indicating that higher contents of these compounds are associated with lower EC_50_ values and stronger antioxidant activity. Protocatechuic acid (−0.09) exhibited a negligible correlation. Salviaflaside (0.889) exhibited a positive correlation. These findings suggest that caffeic acid, danshensu, rosmarinic acid, and protocatechualdehyde are the key antioxidants in PV fruit-spikes.

Quantitative analysis identified danshensu, salviaflaside, and rosmarinic acid as the three primary components with markedly different contents between green and red-brown fruit-spikes. Specifically, danshensu and rosmarinic acid were more abundant in green spikes, while salviaflaside was higher in red-brown spikes. Correlation analysis confirmed a strong association between the levels of danshensu/rosmarinic acid and antioxidant activity. This finding is supported by previous reports highlighting their pronounced antioxidant properties [41,42]. Caffeic acid, another well-known antioxidant, was also proved to be a remarkable contributor. These results demonstrate that green fruit-spikes possess superior antioxidant activity, primarily attributable to their higher content of the specific phenolic acids. And during fruit-spike maturation, the gradual decrease in contents of danshensu and rosmarinic acid may contribute to diminished antioxidant activity.

A discrepancy was observed between the two antioxidant assays when assessing the extraction solvents. Water extracts exhibited higher antioxidant activity in the DPPH assay, whereas 75% ethanol extracts were more active in the ABTS assay. This divergence is likely attributable to the distinct reaction mechanisms and solvent environments of the two assays. The DPPH and the ABTS in vitro assays are fundamental for initial antioxidant evaluation. While our results indicate promising antioxidant activity, further validation through cellular or in vivo studies is necessary to fully elucidate the functional advantages of the extracts.

## 4. Conclusions

This study highlights the unexploited potential of green PV fruit-spikes, traditionally considered agricultural waste, as a rich source of bioactive phenolic compounds with significant antioxidant properties. Our comparative analysis demonstrates that green spikes are a richer source of valuable bioactive compounds, including phenolic acids, flavonoids, and triterpenes, and exhibit superior free radical scavenging capacity compared to red-brown ones. Phenolic acids, particularly caffeic acid, danshensu, rosmarinic acid, and protocatechualdehyde, were identified as key contributors to this enhanced antioxidant activity. Meanwhile, comparative analysis of solvent extracts revealed the chemical and antioxidant differences between water and 75% ethanol extracts. These findings indicate the advantages and potential of green spikes for medicinal and functional food applications, providing a foundation for the utilization of green fruit-spikes generated during large-scale harvesting. Future efforts should focus on in vivo validation of the bioactivities and on optimizing extraction strategies for industrial utilization.

## Figures and Tables

**Table 1:** Constituents identified in the fruit-spikes of *Prunella vulgaris* L. (PV).

No.	RT (min)	Formula	Ion Mode	Calculated Mass (*m*/*z*)	Measured Mass (*m*/*z*)	ppm	MS/MS (*m*/*z*)	Identification	G-W	G-E	R-W	R-E
1	0.80	C_7_H_12_O_6_	[M-H]^−^	191.0556	191.0559	1.57	191, 101	Quinic acid	++	++	+	+
2	0.89	C_4_H_6_O_5_	[M-H]^−^	133.0137	133.0134	−2.26	133, 115	Malic acid	+	+	+	+
3	1.15	C_9_H_10_O_5_	[M-H]^−^	197.0450	197.0452	1.01	197, 179, 151, 135, 123	Danshensu	++	++	++	++
4	1.21	C_8_H_8_O_4_	[M-H]^−^	167.0344	167.0351	4.19	167, 123	Vanillic acid	+	+	+	+
5	1.40	C_7_H_6_O_4_	[M-H]^−^	153.0188	153.0202	9.15	153, 109	Protocatechuic acid	+	+	+	+
6	1.48	C_9_H_10_O_4_	[M-H]^−^	181.0501	181.0508	3.87	163	Dihydrocaffeic acid	+	+	+	+
7	1.56	C_16_H_18_O_9_	[M-H]^−^	353.0873	353.0876	0.85	191, 179, 135, 161	Chlorogenic acid	++	++	+	+
8	1.58	C_13_H_24_O_9_	[M-H]^−^	323.1342	323.1346	1.24	323, 179, 135	Unknown	+	+	+	+
9	1.71	C_10_H_8_O_5_	[M-H]^−^	207.0293	207.0299	2.90	207, 163, 135, 119	4-hydroxy-4-(3-hydroxy-phenyl)-2-oxo-but-3-enoic acid isomer	+	+	+	+
10	1.83	C_7_H_12_O_5_	[M-H]^−^	175.0606	175.0603	−1.71	175, 137, 113	Shikimic acid	+	+	+	+
11	1.91	C_7_H_6_O_3_	[M-H]^−^	137.0239	137.0250	8.03	137, 109	Protocatechualdehyde	+	+	+	+
12	2.19	C_9_H_8_O_4_	[M-H]^−^	179.0344	179.0347	1.68	179, 135	Caffeic acid	++	++	++	++
13	2.26	C_18_H_18_O_9_	[M-H]^−^	377.0873	377.0869	−1.06	377, 359, 331, 197, 124	(2*R*)-3-(3,4-dihydroxyphenyl)-2-[(2*R*)-3-(3,4-dihydroxyphenyl)-2-hydroxypropanoyl]oxypropanoic acid	++	++	+	+
14	2.44	C_18_H_18_O_9_	[M-H]^−^	377.0873	377.0869	−1.06	377, 359, 331, 197, 124	(2*R*)-3-(3,4-dihydroxyphenyl)-2-[(2*R*)-3-(3,4-dihydroxyphenyl)-3-hydroxypropanoyl]oxypropanoic acid isomer	++	+	+	−
15	2.59	C_14_H_24_O_10_	[M-H]^−^	351.1291	351.1298	1.99	351, 295, 197, 179, 161	Unknown	+	+	+	+
16	3.02	C_16_H_20_O_10_	[M-H]^−^	371.0978	371.0978	0.00	371, 300, 121	Dihydro Isoferulic Acid 3-O-*β*-*D*-Glucuronide	++	++	++	++
17	3.14	C_20_H_34_O_10_	[M-H]^−^	433.2074	433.2072	1.99	433, 359, 343, 287, 271, 163, 101, 119	Unknown	+	+	+	+
18	3.41	C_9_H_8_O_3_	[M-H]^−^	163.0395	163.0400	3.07	163,101,119	*p*-Coumaric acid	+	+	+	+
19	3.64	C_27_H_30_O_16_	[M-H]^−^	609.1456	609.1452	−0.66	609, 300	Quercetin 3-O-neohesperidoside	++	++	++	++
20	3.81	C_27_H_30_O_16_	[M-H]^−^	609.1456	609.1452	−0.66	609, 300	Rutin	++	++	++	++
21	4.07	C_21_H_20_O_12_	[M-H]^−^	463.0877	463.0875	−0.66	463, 300, 271, 255, 243, 178, 151	Hyperoside	++	++	++	++
22	4.29	C_21_H_20_O_12_	[M-H]^−^	463.0877	463.0875	−0.43	463, 300, 271, 255, 243, 151	Isoquercitrin	++	++	++	++
23	5.03	C_24_H_26_O_13_	[M-H]^−^	521.1295	521.1288	−1.34	521, 359, 179, 161, 135	Salviaflaside	++	++	++	++
24	5.19	C_27_H_30_O_15_	[M-H]^−^	593.1506	593.1511	0.84	284, 285	Quercetin 3,7-di-O-rhamnoside	+	+	+	+
25	5.41	C_26_H_24_O_12_	[M-H]^−^	527.1190	527.1190	0.00	285, 241, 197, 179, 161	Unknown	++	+	++	+
26	5.65	C_36_H_30_O_16_	[M-H]^−^	717.1456	717.1443	−1.81	717, 519, 339, 321	Isosalvianolic acid E	+	+	+	+
27	5.71	C_21_H_20_O_11_	[M-H]^−^	447.0927	447.0936	2.01	285, 284, 255, 227	KaeMpferol 3-O-*D*-glucopyranoside	+	+	+	+
28	5.86	C_36_H_30_O_16_	[M-H]^−^	717.1456	717.1443	−1.81	229	Salvianolic acid B isomer	+	+	+	+
29	6.08	C_36_H_32_O_16_	[M-H]^−^	719.1612	719.1595	−2.36	719, 673, 539, 197, 179	Rashomonic Acid C/D	++	++	++	++
30	6.52	C_9_H_16_O_4_	[M-H]^−^	187.0973	187.0972	−0.53	187, 171, 141	Unknown	+	++	+	++
31	7.09	C_18_H_16_O_8_	[M-H]^−^	359.0767	359.0772	1.39	719, 359, 179, 161, 135	Rosmarinic acid	++	++	++	++
32	7.60	C_26_H_22_O_10_	[M-H]^−^	493.1135	493.1137	0.41	493, 313, 295, 203, 109	Salvianolic acid A	+	+	+	+
33	7.74	C_26_H_24_O_11_	[M-H]^−^	511.1240	511.1237	−0.59	511, 359, 311, 299, 179	Unknown	++	+	++	+
34	8.10	C_36_H_30_O_17_	[M-H]^−^	733.1405	733.1387	−2.46	733, 553, 509, 373, 329, 179	3-Benzofurancarboxylic acid, 4-[(1*Z*)-3-[(1*R*)-1-carboxy-2-(3,4-dihydroxyphenyl)ethoxy]-2-hydroxy-3-oxo-1-propen-1-yl]-2-(3,4-dihydroxyphenyl)-2,3-dihydro-7-hydroxy-, 3-[(1*R*)-1-carboxy-2-(3,4-dihydroxyphenyl)ethyl] ester, (2*S*,3*S*) isomer	++	++	+	+
35	8.30	C_36_H_30_O_17_	[M-H]^−^	733.1405	733.1387	−2.46	733, 553, 535, 509, 373, 329, 269, 179	Unknown	+	+	++	++
36	8.40	C_16_H_26_O_7_	[M-H]^−^	329.1600	329.1600	0.00	329, 242, 224, 197, 161	Unknown	+	+	+	+
37	8.45	C_37_H_60_O_13_	[M-H]^−^	711.3956	711.3940	−2.25	711, 503, 485	Olean-12-en-28-oic acid, 23-(glycero-manno-heptonoyloxy)-2,3,19-trihydroxy-, (2*α*,3*β*,4*β*,19*α*)-isomer	+	+	++	++
38	8.58	C_18_H_16_O_7_	[M-H]^−^	343.0818	343.0814	−1.17	343, 197, 181, 161, 145	Eupatorin isomer	++	++	+	+
39	8.68	C_27_H_22_O_12_	[M-H]^−^	537.1033	537.1034	0.19	537, 493, 295	Salvianolic acid H	+	++	+	+
40	8.99	C_36_H_28_O_16_	[M-H]^−^	715.1299	715.1283	−2.24	715, 357, 339, 311, 197, 179, 161	3-[(1*R*)-1-Carboxy-2-(3,4-dihydroxyphenyl)ethyl] 5-[(1*E*)-3-[(1*R*)-1-carboxy-2-(3,4-dihydroxyphenyl)ethoxy]-3-oxo-1-propen-1-yl]-2-(3,4-dihydroxyphenyl)-7-hydroxy-3-benzofurancarboxylate	++	++	++	++
41	9.13	C_36_H_30_O_16_	[M-H]^−^	717.1456	717.1435	−2.93	717, 519, 339, 311	Salvianolic acid B	++	++	+	++
42	9.21	C_36_H_28_O_16_	[M-H]^−^	715.1299	715.1283	−2.24	715, 553, 329, 267, 197, 179, 161, 135	Dedihydro-salvianolic acid B isomer	++	++	++	++
43	9.22	C_36_H_30_O_16_	[M-H]^−^	717.1456	717.1435	−2.93	717, 339, 311	Iso salvianolic acid B	++	++	++	++
44	9.26	C_42_H_68_O_15_	[M+HCOO]^−^	857.4535	857.4515	−2.33	857, 811, 503	Tetrahydroxy-ursolic acid-GlcA-Xyl	+	+	+	+
45	9.49	C_20_H_20_O_11_	[M-H]^−^	435.0927	435.0925	−0.46	435, 161	5-O-*β*-*D*-(6′-salicylyl)-glucopyranoside	+	+	+	+
46	9.78	C_36_H_28_O_16_	[M-H]^−^	715.1299	715.1283	−2.24	715, 535, 491, 311, 293, 179	Dedihydro-salvianolic acid B isomer	++	++	++	++
47	9.99	C_36_H_30_O_16_	[M-H]^−^	717.1456	717.1443	−1.81	717, 519, 357, 339, 311, 197, 180	Salvianolic acid E/L	+	+	+	+
48	10.07	C_17_H_14_O_6_	[M-H]^−^	313.0712	313.0710	−0.64	313, 269, 243, 161	Unknown	−	+	−	+
49	10.17	C_62_H_98_O_31_	[M-H]^−^	1337.6014	1337.5930	−6.28	1205, 795, 487	Trihydroxy-ursolic acid-Ara-Ara-Ara-Rha-Xyl-GlcA	++	++	++	++
50	10.37	C_15_H_10_O_6_	[M-H]^−^	285.0399	285.0392	−2.46	285, 267, 257, 241, 135	Luteolin	+	+	+	+
51	10.39	C_57_H_90_O_27_	[M-H]^−^	1205.5591	1205.5527	−5.31	1205, 1073, 941, 663, 487	Trihydroxy-ursolic acid/oleanic acid-Ara-Ara-Ara-Rha-GlcA	++	++	++	++
52	10.55	C_57_H_90_O_26_	[M-H]^−^	1189.5642	1189.5573	−5.80	1189, 1057, 779, 487	Trihydroxy-ursolic acid/oleanic acid-Ara-Rha-Ara-Rha-Rha	++	++	++	++
53	10.56	C_52_H_82_O_23_	[M-H]^−^	1073.5169	1073.5101	−6.33	1073.941, 795, 663, 487	Trihydroxy-ursolic acid/oleanic acid-Ara-Rha-Ara-GlcA	++	++	++	++
54	10.70	C_60_H_92_O_30_	[M-H]^−^	1291.5595	1291.5524	−5.50	1291, 1247, 1115, 927, 795, 663, 487	Unkown	++	++	++	++
55	10.77	C_57_H_88_O_27_	[M-H]^−^	1203.5435	1203.5449	1.16	1087, 941, 795, 663, 487	Trihydroxy-ursolic acid/oleanic acid-Rha-Rha-Ara-GlcA	+	+	+	+
56	11.04	C_58_H_92_O_26_	[M-H]^−^	1203.5799	1203.5707	−7.64	1203, 1071, 647, 471	Dihydroxy-ursolic acid/oleanic acid-Ara-Ara-Rha-Rha-GlcA	++	++	++	++
57	11.17	C_53_H_84_O_23_	[M-H]^−^	1087.5325	1087.5249	−6.99	1087, 795, 663, 487	Trihydroxy-ursolic acid/oleanic acid-Rha-Rha-Ara-GlcA	+	++	++	++
58	11.25	C_61_H_94_O_30_	[M-H]^−^	1305.5752	1305.5658	−7.20	1305, 1261, 1129, 983, 809, 663, 487	Olean-12-ene-23,28-dioic acid, 3-(*β*-*D*-glucopyranuronosyloxy)-2-hydroxy-, 28-(O-*D*-apio-*β*-*D*-furanosyl-(1→3)-O-[O-*β*-*D*-glucopyranosyl-(1→3)-6-deoxy-*α*-*L*-mannopyranosyl-(1→2)]-4-O-acetyl-6-deoxy-βD-galactopyranosyl) ester, (2β,3β,4α)-	+	++	++	++
59	11.58	C_35_H_28_O_14_	[M-H]^−^	671.1401	671.1381	−2.98	671, 487, 329,	Unknown	+	+	+	+
60	11.65	C_53_H_84_O_22_	[M-H]^−^	1071.5376	1071.5284	−8.59	1071, 939, 647, 487	Trihydroxy-ursolic acid/oleanic acid-Ara-Rha-Rha-Ara	+	+	+	+
61	12.00	C_61_H_94_O_29_	[M-H]^−^	1289.5803	1289.5695	−8.59	1289, 1245, 1129, 939, 647	Dihydroxy-ursolic acid/oleanic acid-Ara-Glc-Rha-Rha-GlcA	+	+	+	+
62	12.02	C_57_H_90_O_26_	[M-H]^−^	1189.5642	1189.5546	−8.07	1189, 1057, 779, 471	Dihydroxy-ursolic acid/oleanic acid-Ara-Ara-Rha-Rha-Glc	++	++	++	++
63	12.33	C_57_H_90_O_26_	[M-H]^−^	1189.5642	1189.5546	−8.07	1189, 1057, 925, 779, 617, 471	Dihydroxy-ursolic acid/oleanic acid-Ara-Ara-Rha-Rha-Glc	++	++	++	++
64	12.71	C_41_H_64_O_15_	[M-H]^−^	795.4167	795.4131	−4.53	795, 487	Trihydroxy-ursolic acid/oleanic acid-Ara-GlcA	++	++	++	++
65	12.73	C_52_H_82_O_22_	[M-H]^−^	1057.5219	1057.5149	−6.62	1057, 925, 647, 471	Dihydroxy-ursolic acid/oleanic acid-Ara-Ara-Rha-GlcA	++	++	++	++
66	12.93	C_53_H_84_O_22_	[M-H]^−^	1071.5376	1071.5298	−7.28	1071, 647, 471	Dihydroxy-ursolic acid/oleanic acid-Ara-Rha-Rha-GlcA	+	+	+	+
67	13.26	C_60_H_92_O_29_	[M-H]^−^	1275.5705	1275.5569	−10.66	1275, 1231, 1099, 793, 647, 471	Unknown	+	+	+	+
68	13.69	C_44_H_34_O_18_	[M-H]^−^	849.1667	849.1633	−4.00	359	Unknown	+	+	+	+
69	13.77	C_36_H_56_O_11_	[M-H]^−^	663.3744	663.3726	−2.71	663, 487	Trihydroxy-ursolic acid/oleanic acid-GlcA	+	+	+	+
70	14.04	C_18_H_28_O_4_	[M-H]^−^	307.1909	307.1902	−2.28	307,266,179,161	Unknown	+	+	+	+
71	14.07	C_54_H_84_O_23_	[M-H]^−^	1099.5325	1099.5338	1.20	1099, 487	Olean-12-en-28-oic acid, 3-(*β*-*D*-glucopyranosyloxy)-2,23-dihydroxy-16-oxo-, O-*β*-*D*-xylopyranosyl-(1→4)-O-6-deoxy-*α*-*L*-mannopyranosyl-(1→2)-6-deoxy-*β*-*D*-galactopyranosyl ester, (2*β*,3*β*,4*α*)-(9CI)	+	+	+	+
72	14.11	C_44_H_34_O_18_	[M-H]^−^	849.1667	849.1633	−4.00	849, 593, 359	Unknown	+	++	+	+
73	14.10	C_30_H_48_O_6_	[M-H]^−^	503.3373	503.3360	−2.58	549 ([M+HCOO]^−^), 503, 485	Tetrahydroxyurs-12-en-28-oic acid	+	+	+	+
74	14.22	C_51_H_82_O_21_	[M+HCOO]^−^	1075.5325	1073.5222	−9.58	1029, 941, 779, 647, 633, 487	Unknown	+	+	+	+
75	14.22	C_51_H_80_O_21_	[M+HCOO]^−^	1073.5169	1073.5074	−8.85	1027, 943, 633, 487	Unknown	+	+	+	+
76	14.32	C_34_H_26_O_12_	[M-H]^−^	625.1346	625.1342	−0.64	625, 501, 471, 366, 135	Unknown	+	+	+	+
77	14.34	C_18_H_26_O_4_	[M-H]^−^	305.1753	305.1750	−0.98	305, 290, 274, 161	Unknown	+	+	+	+
78	14.50	C_30_H_46_O_5_	[M-H]^−^	485.3267	485.3264	−0.62	485, 458	2*α*,3*α*,24-trihydroxylolean-11,13 (18)-dien-28-oic acid isomer	+	+	++	++
79	14.54	C_18_H_30_O_4_	[M-H]^−^	309.2066	309.2073	2.30	309, 266, 216	Unknown	++	+	++	+
80	14.65	C_30_H_48_O_5_	[M-H]^−^	487.3423	487.3422	−0.21	487, 469	Trihydroxy-oleanolic acid isomer	+	++	+	++
81	14.72	C_30_H_48_O_5_	[M-H]^−^	487.3423	487.3422	−0.21	487	Trihydroxy-oleanolic acid	+	++	+	++
82	14.82	C_40_H_62_O_13_	[M-H]^−^	749.4112	749.4100	−1.60	749, 633, 487	Unknown	+	+	++	++
83	15.13	C_34_H_42_O_9_	[M-H]^−^	593.2751	593.2725	−4.38	593, 487, 378, 304, 290, 216	Unknown	+	++	+	++
84	15.27	C_30_H_48_O_5_	[M-H]^−^	487.3423	487.3422	−0.21	487	Trihydroxy-ursolic acid	+	++	+	++
85	15.33	C_30_H_48_O_5_	[M-H]^−^	487.3423	487.3422	−0.21	487	Trihydroxy-ursolic acid isomer	+	++	+	++
86	15.43	C_18_H_30_O_3_	[M-H]^−^	293.2117	293.2110	−2.39	293, 277, 275, 225, 183, 130	Unknown	+	++	++	++
87	15.46	C_30_H_42_O_11_	[M-H]^−^	577.2649	577.2678	5.02	577, 277, 225	Unknown	+	++	+	++
88	15.46	C_35_H_54_O_9_	[M-H]^−^	617.3690	617.3683	−1.13	617, 441, 223	Prunelloside A	+	++	++	++
89	15.65	C_30_H_46_O_4_	[M-H]^−^	469.3318	469.3311	−1.49	469, 424	2*α*,3*α*-dihydroxylurs-12,20 (30)-dien-28-oic acid	+	++	+	++
90	15.79	C_34_H_44_O_9_	[M-H]^−^	595.2907	595.2888	−3.19	595, 580, 564, 476	Unknown	+	++	++	++
91	15.86	C_28_H_48_O_11_	[M-H]^−^	559.3118	559.3117	−0.18	559, 471, 277	Unknown	+	++	+	++
92	15.90	C_30_H_48_O_4_	[M-H]^−^	471.3474	471.3473	−0.21	471	Dihydroxy-oleanolic acid isomer	+	++	+	++
93	16.00	C_30_H_48_O_4_	[M-H]^−^	471.3474	471.3473	−0.21	517 ([M+HCOO]^−^), 471	Dihydroxy-oleanolic acid isomer	+	+	+	+
94	16.13	C_18_H_32_O_4_	[M-H]^−^	295.2273	295.2266	−2.37	295, 277, 221, 152	*α*-Hydroxylinoleic acid	+	++	+	++
95	16.23	C_30_H_48_O_4_	[M-H]^−^	471.3474	471.3473	−0.21	517 ([M+HCOO]^−^), 471	Dihydroxy-oleanolic acid	+	++	+	++
96	16.29	C_18_H_30_O_3_	[M-H]^−^	293.2117	293.2110	−2.39	293, 277, 225, 180	Unknown	+	+	+	++
97	16.40	C_30_H_48_O_4_	[M-H]^−^	471.3474	471.3473	−0.21	517 ([M+HCOO]^−^), 471	Dihydroxy-ursolic acid isomer	+	++	+	++
98	16.59	C_18_H_30_O_3_	[M-H]^−^	293.2117	293.2108	−3.07	293, 279, 255, 152	Unknown	+	++	+	++
99	17.07	C_28_H_44_O_11_	[M-H]^−^	555.2805	555.2834	5.22	555, 514, 483, 327, 281	Unknown	+	++	+	++
100	17.30	C_30_H_48_O_4_	[M-H]^−^	471.3474	471.3473	−0.21	517 ([M+HCOO]^−^), 471	Dihydroxy-ursolic acid	+	++	+	++
101	17.74	C_39_H_54_O_6_	[M-H]^−^	617.3842	617.3823	−3.08	617, 581, 145	Unknown	+	+	+	++
102	18.50	C_30_H_48_O_3_	[M-H]^−^	455.3525	455.3515	−2.20	455	Betulinic acid	+	+	++	++
103	18.80	C_18_H_30_O_2_	[M-H]^−^	277.2168	277.2166	−0.72	277, 116	Linolenic acid	++	++	++	++
104	18.83	C_30_H_48_O_3_	[M-H]^−^	455.3525	455.3515	−2.20	455	Oleanolic acid	++	++	++	++
105	19.00	C_30_H_48_O_3_	[M-H]^−^	455.3525	455.3515	−2.20	455	Ursolic acid	++	++	+	++
106	19.96	C_18_H_32_O_2_	[M-H]^−^	279.2324	279.2322	−0.72	279, 152, 116	Linoleic acid	+	++	++	++

G, green fruit-spikes; R, red-brown fruit-spikes; W, water extract; E, 75% ethanol extract; ++, the bpi graph shows a peak; +, the extracted ions can be detected; −, below the detection limit.

**Table 2:** Contents of six phenolic acids between green and red-brown fruit-spikes (mg/g).

Sample	Danshensu	Protocatechuic Acid	Protocatechualdehyde	Caffeic Acid	Salviaflaside	Rosmarinic Acid	Total
G-W	3.1 ± 0.0645 ^a^	0.0466 ± 0.00274 ^a^	0.074 ± 0.00204 ^a^	0.65 ± 0.0149 ^a^	0.232 ± 0.00867 ^a^	17.3 ± 0.387 ^a^	21.4 ± 0.442 ^a^
G-E	1.59 ± 0.0589 ^b^	0.02061 ± 0.0003 ^b^	0.0455 ± 0.00138 ^b^	0.417 ± 0.0119 ^b^	0.281 ± 0.00502 ^b^	21.6 ± 0.168 ^b^	24.0 ± 0.228 ^b^
R-W	1.68 ± 0.0037 ^b^	0.0729 ± 0.00128 ^c^	0.076 ± 0.00213 ^a^	0.478 ± 0.0093 ^c^	0.665 ± 0.0247 ^c^	3.68 ± 0.113 ^c^	6.65 ± 0.148 ^c^
R-E	0.86 ± 0.0127 ^c^	0.0362 ± 0.000726 ^a^	0.0462 ± 0.00188 ^b^	0.302 ± 0.0106 ^d^	0.888 ± 0.0229 ^d^	4.8 ± 0.108 ^d^	6.93 ± 0.148 ^d^

G, green fruit-spikes; R, red-brown fruit-spikes; W, water extract; E, 75% ethanol extract. The difference in superscript letters in a column indicates a significant difference according to Dunnett’s T3 multiple-comparison test for protocatechuic acid contents or Tukey’s post hoc test for others (*p* ≤ 0.05).

**Table 3:** EC_50_ of DPPH and ABTS between green and red-brown fruit-spikes (g/L).

Compounds	DPPH	ABTS
G-W	0.341 ± 0.0221 ^a^	2.84 ± 0.191 ^a^
G-E	0.605 ± 0.0464 ^b^	2.60 ± 0.181 ^a^
R-W	0.659 ± 0.0366 ^b^	8.99 ± 0.856 ^b^
R-E	0.887 ± 0.0527 ^c^	7.62 ± 0.273 ^b^

G, green fruit-spikes; R, red-brown fruit-spikes; W, water extract; E, 75% ethanol extract. The difference in superscript letters in a column indicates a significant difference according to Dunnett’s T3 multiple-comparison test (*p* ≤ 0.05).

## Data Availability

Data is contained within the article.

## Abbreviations

The following abbreviations are used in this manuscript:

ABTS	2,2′-azino-bis (3-ethylbenzothiazoline-6-sulfonic acid) diammonium salt
CHP	*Chinese Pharmacopoeia*
EC_50_	Half-Maximal Effective Concentration
DPPH	1-diphenyl-2-picrylhydrazine
HPLC	High-Performance Liquid Chromatography
PV	*Prunella vulgaris* L.
TIC	Total-Ion Chromatogram
UPLC	Ultra-High-Performance Liquid Chromatography
ESI-Q-TOF-MS	Electrospray Ionization–Quadrupole Time-of-Flight Mass Spectrometry
